# Supplementary material for: Private QTLs and the genetic architecture of hierarchical size traits: from body size to sex-specific plasticity
Source: G3 (Bethesda). 2026 Apr 18;16(6):jkag096. doi: 10.1093/g3journal/jkag096 (PMC13232497; doi:10.1093/g3journal/jkag096)
Supplement: jkag096_Supplementary_Data [file jkag096_supplementary_data.zip › Supplemental_Material_Legends_G3-2026-406691.docx]

## Supplementary Material

**Supplementary Figure 1.** Overlap of candidate SNPs (red) and genes (black) for different size-related phenotypes. (A) Overlap between male, female and sex-averaged body size and SSD in starved flies. (B) Overlap in male, female, and sex-averaged body size and SSD between fed and starved flies. Candidate SNPs were identified by MLM GWAS and candidate genes were identified by VEGAS.

**Supplementary Figure 2.** The accumulation of MLM GWAS hits (*p*<1x10^-5^) with the inclusion of increasingly large number of SNPs, ranked by their DGRP2 GWAS *p-*value. For most traits, the accumulation plateaus after ~4,000 SNPs, suggesting that conducting an MLM GWAS on the 10,000 SNPs with the lowest DGPR2 GWAS *p-*value provides a sufficiently inclusive set to capture all MLM GWAS hits.

**Supplementary Table 1.** SNP and Gene hits for MLM GWAS and VEGAS applied to fed and starved female and male body size, female and male plasticity, fed and starved SSD, and SSP (p<1x10^-5^).

**Supplementary Table 2.** Results of GO and KEGG analysis on candidate genes for body size, plasticity and SSP.

**Supplementary Table 3.** List of focal growth-regulatory and nutrient-signaling genes.

**Supplementary Table 4.** Results of VEGAS for SSP, fed SSD and fed sex-averaged body size, focused on growth-regulatory genes and nutrient-sensing genes (see Supplementary Table 3). Each table is labelled by phenotype (SSP, fed SSD and fed sex-averaged body size) and the gene set being evaluated (growth-regulatory genes or nutrient-sensing genes).

**Supplementary Table 5:** Stocks used for the functional validation
