# Supplementary material for: Private QTLs and the genetic architecture of hierarchical size traits: from body size to sex-specific plasticity
Source: G3 (Bethesda). 2026 Apr 18;16(6):jkag096. doi: 10.1093/g3journal/jkag096 (PMC13232497; doi:10.1093/g3journal/jkag096)
Supplement: jkag096_Supplementary_Data [file jkag096_supplementary_data.zip › Supplementary_Figures_G3-2026-406691.pdf]

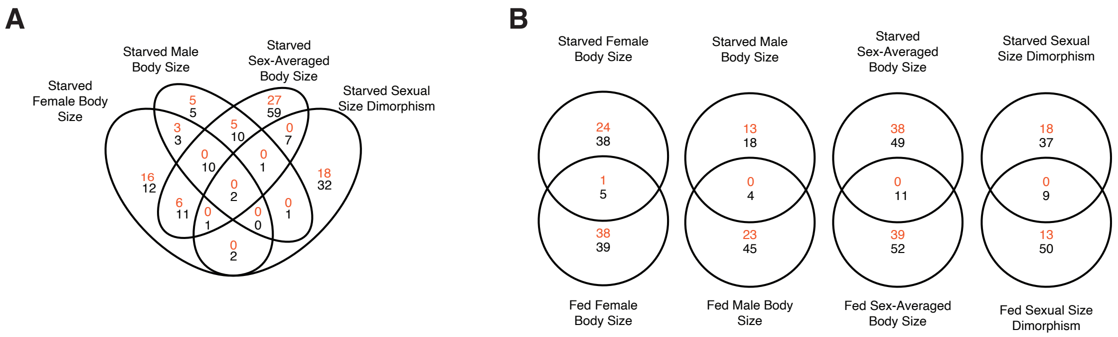

Supplementary Figure 1. Overlap of candidate SNPs (red) and genes (black) for different size-related phenotypes. (A) Overlap between male, female and sex-averaged body size and SSD in starved flies. (B) Overlap in male, female, and sex-averaged body size and SSD between fed and starved flies. Candidate SNPs were identified by MLM GWAS and candidate genes were identified by VEGAS.

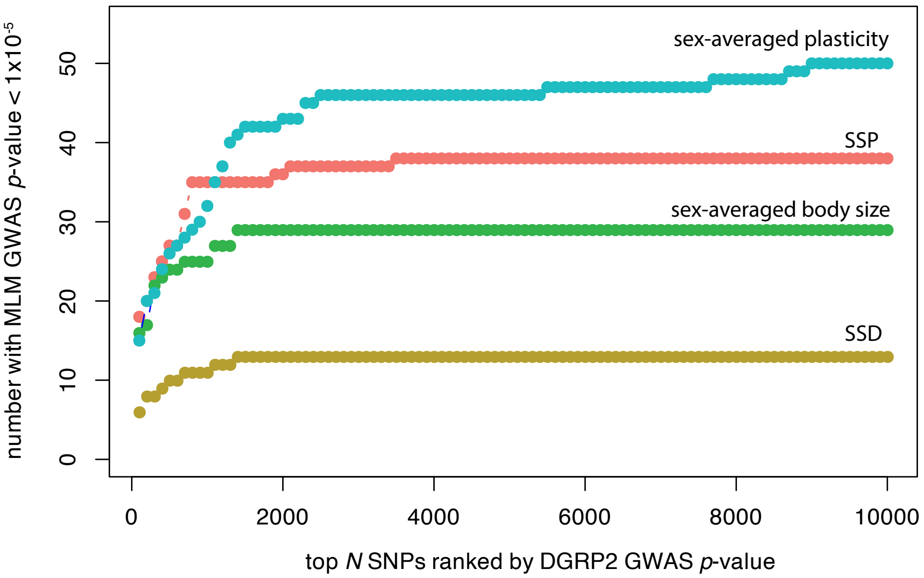

Supplementary Figure 2. The accumulation of MLM GWAS hits with the inclusion of increasingly large number of SNPs, ranked by their DGRP2 GWAS *p*-value. For most traits, the accumulation plateaus after ~4,000 SNPs, suggesting that conducting an MLM GWAS on the 10,000 SNPs with the lowest DGPR2 GWAS *p*-value provides a sufficiently inclusive set to capture all MLM GWAS hits ($p<1\times10^{-5}$).
